# Chest wall lymph node metastasis from follicular thyroid carcinoma: a rare case report

**DOI:** 10.1186/s13000-019-0907-0

**Published:** 2019-11-20

**Authors:** Taolang Li, Zhiyuan Ma, Chengli Lu, Quanzhong Zhou, Zelong Feng, Xinglong Wu, Yi Luo, Dan Li, Xiaoming Cheng, Xuemei Liu

**Affiliations:** 1 Thyroid and Breast Surgery Department, The First Affiliated Hospital of Zunyi Medical University, Zunyi, China; 2 Imaging Department, The First Affiliated Hospital of Zunyi Medical University, Zunyi, China; 3 Nuclear Medicine Department, The First Affiliated Hospital of Zunyi Medical University, Zunyi, China; 4 Pathology Department, The First Affiliated Hospital of Zunyi Medical University, Zunyi, China; 5 Gastroenterology Department, The First Affiliated Hospital of Zunyi Medical University, Zunyi, China

**Keywords:** Follicular thyroid carcinoma, Metastasis, Lymph node, Chest wall, Papillary thyroid cancer

## Abstract

**Background:**

Distant metastases from follicular thyroid carcinoma are mainly hematogenous and are commonly observed in the lungs and bones. Other rare sites are the parotid gland, skin, brain, ovary, adrenal gland, kidney, pancreas and breast, with chest wall lymph node metastasis being even more rare.

**Case presentation:**

Over the past 10 years, three surgeries were performed on a 69-year-old women with a history of follicular thyroid cancer and its metastatic lesions. The patient presented with a 3-month history of masses in the left chest. She underwent detailed examination of the chest wall tumors, and surgery was then performed to resect all of the tumors. Based on the histopathology, these lymph nodes were confirmed to harbor metastatic follicular thyroid carcinoma.

**Conclusion:**

This study reports the first case of follicular thyroid carcinoma metastasis to the chest wall lymph node.

## Background

Follicular thyroid carcinoma (FTC) is the second most common thyroid cancer subtype after papillary thyroid cancer (PTC), and it accounts for approximately 10% of all thyroid cancers [1]. The average prognosis of FTC is worse than that of PTC [2]. Follicular thyroid cancer is known to metastasize hematogenously, which could explain its more common distant spread compared with papillary thyroid cancer [3]. The incidence of distant metastasis in FTC has been reported to be 6–20% [4–6]. The bones and lungs are the most frequent sites of distant metastases. However, metastases can also occur in some unusual sites (e.g., the parotid gland, skin, brain, ovary, adrenal gland, kidney, pancreas, breast and eye) [3]. Cervical lymph node metastases at diagnosis are significantly less common in follicular thyroid cancer than in papillary thyroid cancer, with an incidence of 2–8% [7, 8]; this rate increases to 17% when widely invasive follicular thyroid cancer is considered [9]. However, distant lymph node metastases have been reported sporadically, with a few reports of axillary lymph node metastases [10, 11]. This study contributes to the literature one additional case of chest wall lymph node metastasis from follicular thyroid carcinoma in a 69-year-old woman.

## Case presentation

**Fig. 1:**
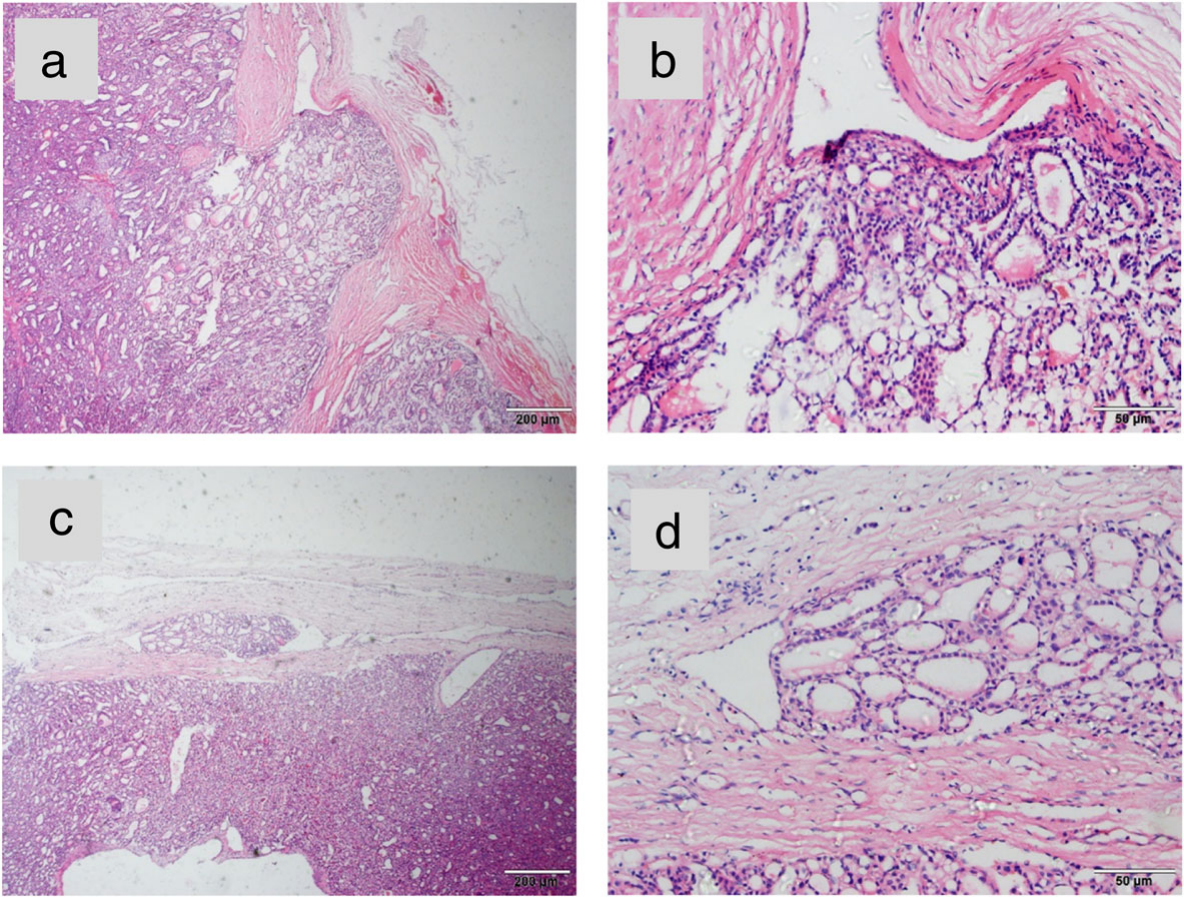
Tumor tissue infiltration was observed in the local capsule (**a**) and (**b**), and tumor tissue invasion was observed in the capsule vessels(**c**) and (**d**). (Original magnification qre 40x in A and C, 200x in B and D)

**Fig. 2:**
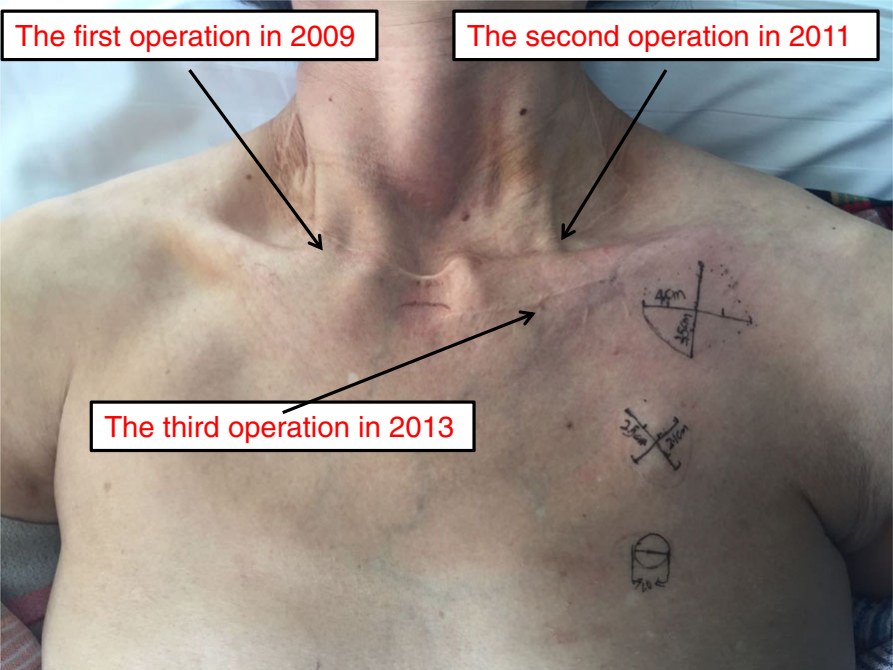
Masses observed in the left chest wall. The largest one was in the left subclavian area, with a size of 4 × 3.5 cm; the intermediate and smallest masses were located in the second and third intercostal levels and were 2.5 × 2 cm and 1 × 1 cm in size, respectively. Scars from the previous three operations are clearly visible

**Fig. 3:**
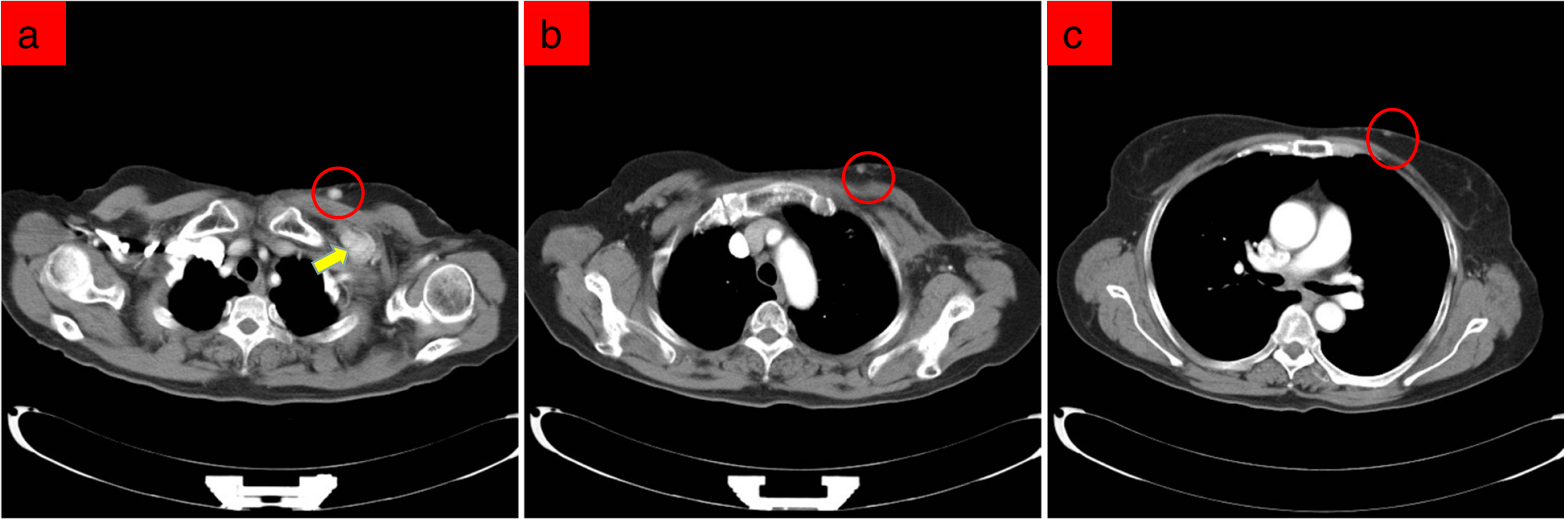
CT scan showing the shadow of the horizontal cutaneous nodules (red circle) in the left subclavian region (**a**) and the second (**b**) and third (**c**) intercostal spaces of the left anterior chest wall. The largest one is in the left subclavian area, which measured approximately 3 × 3 cm (yellow arrow); the density was uniform, the boundary was clear, the enhancement scan was non-uniform and significantly enhanced, and lymph node metastasis was considered. A B

**Fig. 4:**
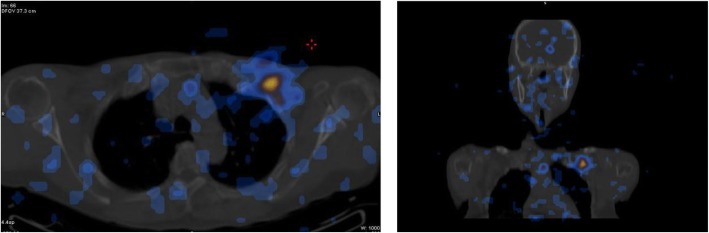
Systemic and local scans were performed at 48 h after treatment with oral I131 5 mCi. No thyroid development was observed. The left subclavian reflex concentration is considered thyroid cancer metastasis to the left subclavian lymph node

**Fig. 5:**
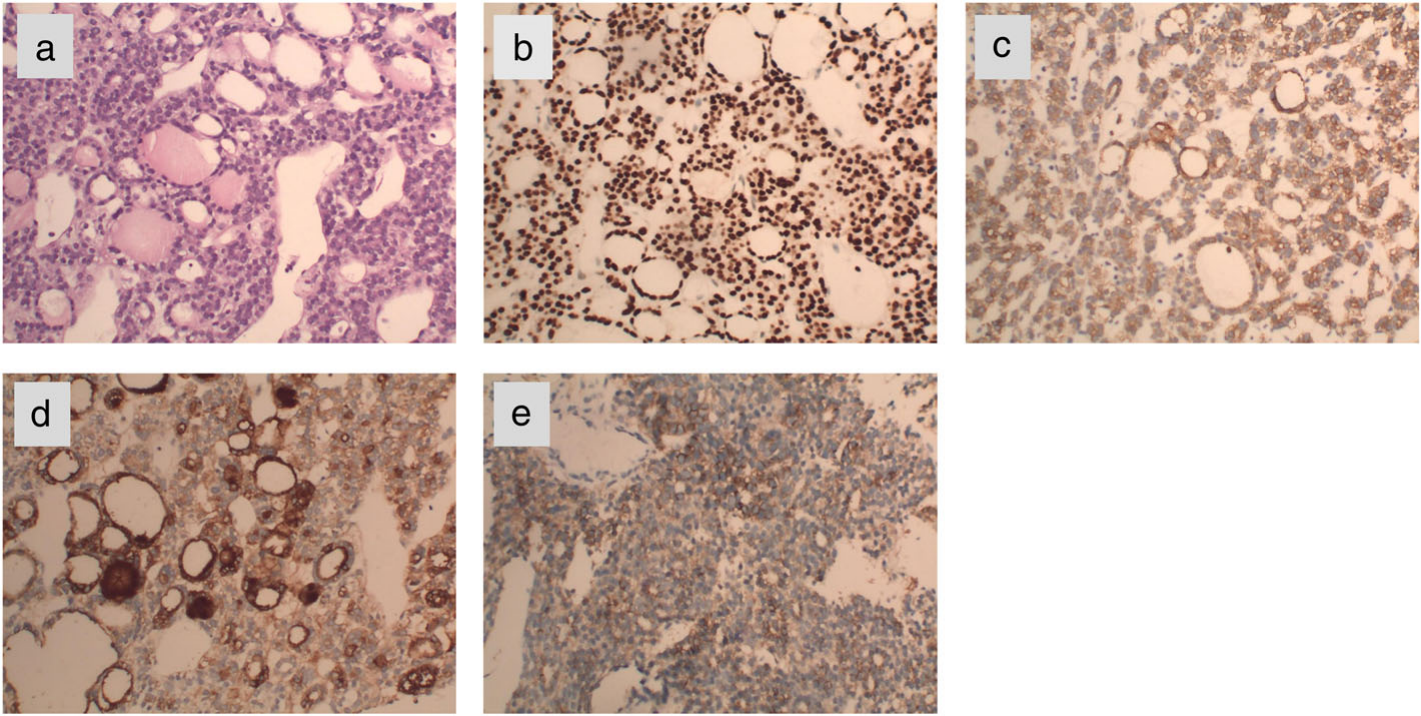
The tumor cells were mildly dysplastic and formed follicular structures of different sizes. Glial secretions were seen in some follicular cavities(**a**), Immunohistochemical staining for TTF1(**b**), CK7(**c**), thyroglobulin(**d**) and CD56(**e**) were positive. (original magnification, 200x)

**Fig. 6:**
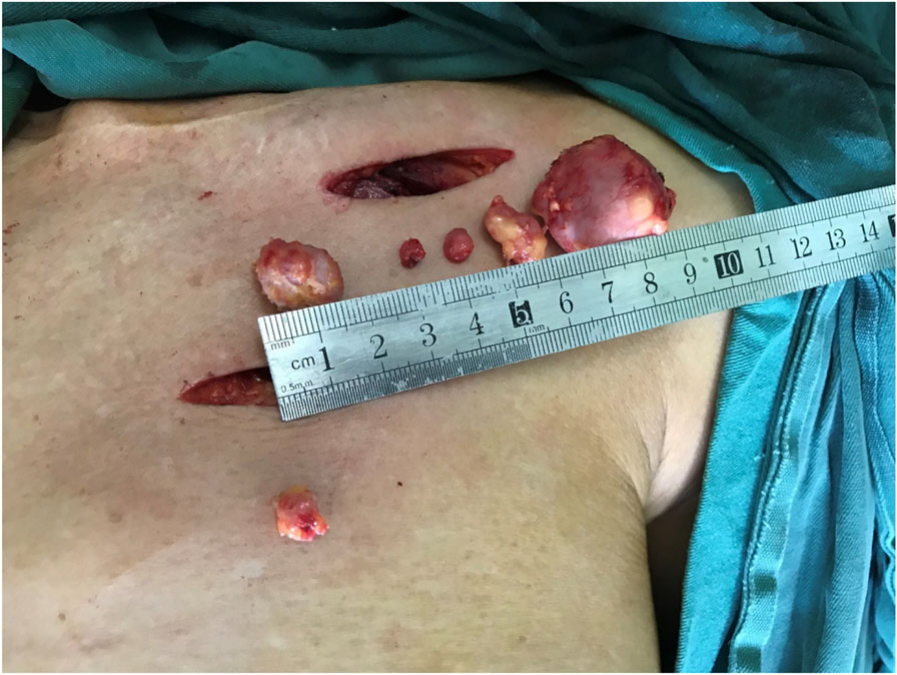
A total of 4 lymph nodes were removed from under the left clavicle with a maximum diameter of 3 cm. The second intercostal lymph node was 1.5 cm in diameter; the diameter of the third intercostal lymph node was 0.6 cm, and it was completely removed through the second incision

**Fig. 7:**
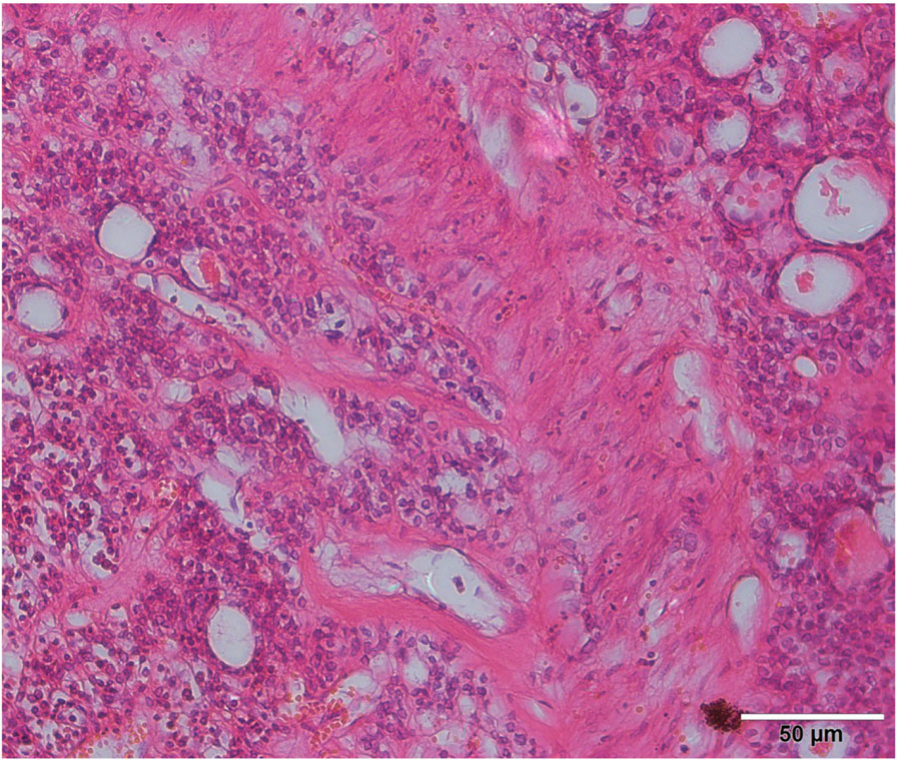
In the lymph node, dysplastic cells were found to be arranged in a microfollicular or trabecular shape; no papillary structure, no obvious increase in cell volume, and no obvious nuclear pseudoinclusion body were observed (hematoxylin-eosin staining; original magnification, 200x)

In March 2018, a 69-year-old woman with a history of follicular thyroid cancer presented with a 3-month history of masses in the left chest. She had undergone total thyroidectomy with right-sided cervical lymphadenectomy in our department and pathological diagnosis was thyroid follicular carcinoma (Fig. 1), followed postoperative iodine treatment 10 years previously. Two years later, the patient developed a recurrence of left cervical lymphadenopathy, for which she underwent left-sided modified radical neck dissection. Unfortunately, she experienced left supraclavicular and left axillary lymph node recurrence in 2013 and underwent a third operation to resect these metastatic lesions. Following the third iodine treatment, levothyroxine tablets were administered. Physical examination revealed solid, mobile, painless masses in the left chest wall, the largest of which was approximately 4 × 3.5 cm in size below the left clavicle; a medium-sized mass was found in the second intercostal space that was 2.5 × 2 cm in size, while the smallest mass was in the third intercostal space and measured 1 × 1 cm in size (Fig. 2). Ultrasonographic examination revealed hypoechoic lymph nodes. A computed tomography (CT) scan of the chest showed lymph node enlargement in the subclavian area, and no pulmonary metastases were found (Fig. 3). Systemic and local scans were performed 48 h after oral administration of iodine-131 5 mCi. There was no significant radioactive concentration in the thyroid region, but there was radioactive concentration under the left clavicle and increased point-like radioactive uptake in the left chest wall. Left subclavian and thoracic lymph node metastases of thyroid carcinoma were considered (Fig. 4). Cranial and abdominal CT and whole-body bone scans did not reveal further pathology. Laboratory test results revealed normal T4 and T3 levels, a thyroid-stimulating hormone (TSH) level of 2.5 pg/ml (normal < 10 pg/ml) and serum thyroglobulin (TG) level of 3000 ng/ml (normal < 50 ng/ml). Ultrasound-guided core-needle biopsy of the mass revealed a follicular thyroid neoplasm with lymph node metastases. The results of immunohistochemical analysis indicated TTF, CK7, and thyroglobulin positivity and partial CD56 positivity, while the test for CgA was negative; the Ki67 index was 5%(Fig. 5). Mass resection was performed on the left chest wall under local anesthesia. Multiple lymph nodes were found to be enlarged under the left clavicle, and a total of 4 lymph nodes were removed, with a maximum diameter of 3 cm. The second intercostal lymph node was 1.5 cm in diameter, and the diameter of the third intercostal lymph node was 0.6 cm (Fig. 6). The final pathological diagnosis was thyroid follicular carcinoma metastasis to the lymph node (Fig. 7). Following a I^131^ radiotherapy session, TSH suppression therapy was continued. Nearly 1 year after the last operation, the patient’s serum thyroglobulin level has remained below the threshold, and no local recurrence or distant metastasis has been observed.

## Discussion

Although follicular thyroid cancer is a relatively indolent differentiated thyroid cancer, distant metastasis may occur in some patients because it tends to invade blood vessels and metastasize by hematogenous spread to distant sites, most commonly to the bones and lungs, although other unusual sites such as the parotid gland, skin, brain, ovary, adrenal gland, kidney, pancreas, breast and eye have been reported in the literature. Cervical lymph node metastases are less common in follicular thyroid carcinoma, and distant lymph node metastasis is very rare. The pathophysiology of metastatic thyroid carcinoma to distant lymph nodes remains elusive [12]. However, the routes of metastatic chest wall tumors are commonly hematogenous, lymphogenous and diaphragmatic [13]. Cervical lymphatics are also in communication with mediastinal lymph nodes, and consequently, mediastinal lymph nodes are occasionally involved in thyroid malignancies [14, 15]. However, CT and iodine-131 whole-body scanning did not show any sign of mediastinal lymph node metastasis in this 69-year-old woman. Rouviere described the communication between cervical and axillary lymphatics in 1932 [16], reporting that the physiologic flow to the jugulo-subclavian junction is centripetal. This centripetal flow can change under certain circumstances, such as when sentinel nodes around the lymphatic terminus in the jugulo-subclavian confluence block lymphatic flow due to tumor invasion, surgical manipulation or radiotherapy. Then, a retrograde flow pattern follows along the transverse cervical lymph node in the supraclavicular region, finally leading to axillary lymph node metastasis. This finding is supported by a review of all known cases of thyroid carcinoma metastasis to the axillary lymph nodes, showing that extensive cervical metastases and/or previous neck dissection may favor lymphatic over hematogenous spread [12]. This finding is also supported by the fact that 2 to 9% of patients have died of other head and neck cancers with axillary node metastases [17, 18]. The natural lymphatic drainage of the patient was disrupted due to previous bilateral cervical dissection and axillary and supraclavicular lymph node dissection; then, the lymphatic flow changed to a retrograde pattern and moved through the subclavian to the chest wall. This may explain the observation of metastatic lymph nodes on the left chest wall that gradually decreased in size from top to bottom.

Survival in FTC cases is associated with older age at the time of diagnosis, larger tumor size, capsular invasion, male sex and the presence of distant metastases [19, 20]. The presence of distant metastases predicts a lower long-term survival [3] and often necessitates additional surgical intervention, external beam radiotherapy, endocrine therapy and radioactive iodine treatment. This finding was also supported by the results of the study by Victor Zaydfudim et al. [21], who queried the Surveillance, Epidemiology, and End Results registry for patients diagnosed with well-differentiated thyroid carcinoma between 1988 and 2003. Cases were stratified by age (< 45 vs ≥ 45 years) and pathology (papillary/follicular). Finally, in patients with FTC, lymph node involvement conferred an increased risk of death in both age groups (*P* ≤ .002). However, Eleonora Farina et al. [22] came to the opposite conclusion, suggesting that metastasis to rare sites does not always represent a negative prognostic factor for disease outcome. In fact, they can occur as single distant lesions, and if surgically resectable, their treatment can also lead to local disease remission. In the past 10 years, our patient with right FTC underwent four operations for lymph node metastasis in the contralateral neck, supraclavicular and axillary lymph nodes, plus the thoracic wall lymph node metastasis described in this case report. The final pathological results confirmed lymph node metastasis from follicular thyroid cancer. At the same time, she underwent several I^131^ radiotherapy sessions, as well as long-term TSH suppression therapy. Nearly 1 year after the last operation, no local recurrence or distant metastasis has been found.

With regard to the reason chest wall metastasis occurred, the failure to completely clean the small lymph nodes below the left clavicle when the upper and left axillary lymph nodes were removed for the third operation may be an explanation; this failure could have opened the possibility for subsequent metastasis to the chest wall. In addition, iodine treatment resistance may also occur. In the future, we should consider performing gene detection and the use of targeted therapy.

## Conclusion

Distant lymph node metastasis from follicular thyroid cancer is very rare. This study is the first report of a case of follicular thyroid carcinoma metastasis to the chest wall lymph node. Active comprehensive treatment, as well as long-term follow-up management, should be provided for these patients.

## Data Availability

Not applicable.

## Abbreviations

CT: Computed tomography
FTC: Follicular thyroid carcinoma
PTC: Papillary thyroid cancer
TG: Thyroglobulin
TSH: Thyroid-stimulating hormone

## References

[1] Grani G, Lamartina L, Durante C, Filetti S, Cooper DS (2018). Follicular thyroid cancer and Hurthle cell carcinoma: challenges in diagnosis, treatment, and clinical management. Lancet Diabetes Endocrinol.

[2] Liu Z, Zeng W, Huang L, Wang Z, Wang M, Zhou L (2018). Prognosis of FTC compared to PTC and FVPTC: findings based on SEER database using propensity score matching analysis. Am J Cancer Res.

[3] Madani A, Jozaghi Y, Tabah R, How J, Mitmaker E (2015). Rare metastases of well-differentiated thyroid cancers: a systematic review. Annal Surg Oncol.

[4] Lin JD, Huang MJ, Juang JH, Chao TC, Huang BY, Chen KW (1999). Factors related to the survival of papillary and follicular thyroid carcinoma patients with distant metastases. Thyroid.

[5] Schlumberger M, Challeton C, De Vathaire F, Travagli JP, Gardet P, Lumbroso JD (1996). Radioactive iodine treatment and external radiotherapy for lung and bone metastases from thyroid carcinoma. J Nucl Med.

[6] Mihailovic J, Stefanovic L, Malesevic M (2007). Differentiated thyroid carcinoma with distant metastases: probability of survival and its predicting factors. Cancer Biother Radiopharm.

[7] Parameswaran R, Shulin Hu J, Min En N, Tan WB, Yuan NK (2017). Patterns of metastasis in follicular thyroid carcinoma and the difference between early and delayed presentation. Ann R Coll Surg Engl.

[8] Oyer SL, Fritsch VA, Lentsch EJ (2014). Comparison of survival rates between papillary and follicular thyroid carcinomas among 36,725 patients. Ann Otol Rhinol Laryngol.

[9] Podda M, Saba A, Porru F, Reccia I, Pisanu A (2015). Follicular thyroid carcinoma: differences in clinical relevance between minimally invasive and widely invasive tumors. World J Surg Oncol.

[10] Chiofalo MG, Losito NS, Fulciniti F, Setola SV, Tommaselli A, Marone U (2012). Axillary node metastasis from differentiated thyroid carcinoma with Hurthle and signet ring cell differentiation. A case of disseminated thyroid cancer with peculiar histologic findings. BMC cancer.

[11] Wei BJ, Shen H, Ge F (2013). Management of thyroid carcinoma with metastases in the upper mediastinum and axillary area. Zhonghua er bi yan hou tou jing wai ke za zhi.

[12] Cummings AL, Goldfarb M (2014). Thyroid carcinoma metastases to axillary lymph nodes: report of two rare cases of papillary and medullary thyroid carcinoma and literature review. Endocr Pract.

[13] Hoshino H, Ishikawa A, Kadoyama C (2013). Metastatic chest wall tumor after resection of primary rectal cancer: A case report. Jpn J Chest Surg.

[14] Nakayama H, Wada N, Masudo Y, Rino Y (2007). Axillary lymph node metastasis from papillary thyroid carcinoma: report of a case. Surgery Today.

[15] Ueda S, Takahashi H, Meihe T (1996). T N. a case of axillary lymph nodes recurrence of papillary carcinoma 7 years after the operation. J Jpn Surg Assoc.

[16] H R (1932). Anatomie des lymphatiques de l’homme.

[17] Oo AL, Yamaguchi S, Iwaki H, Amagasa T (2004). Axillary nodal metastasis from oral and maxillofacial cancers: a report of 3 cases. J Oral Maxillofac Surg.

[18] Ers V, Galant C, Malaise J, Rahier J, Daumerie C (2006). Axillary lymph node metastasis in recurrence of papillary thyroid carcinoma: a case report. Wien Klin Wochenschr.

[19] Zidan J, Kassem S, Kuten A (2000). Follicular carcinoma of the thyroid gland: prognostic factors, treatment, and survival. Am J Clin Oncol.

[20] Baloch ZW, LiVolsi VA (2001). Prognostic factors in well-differentiated follicular-derived carcinoma and medullary thyroid carcinoma. Thyroid.

[21] Zaydfudim V, Feurer ID, Griffin MR, Phay JE (2008). The impact of lymph node involvement on survival in patients with papillary and follicular thyroid carcinoma. Surgery.

[22] Farina E, Monari F, Tallini G, Repaci A, Mazzarotto R, Giunchi F (2016). Unusual Thyroid Carcinoma Metastases: a Case Series and Literature Review. Endocr Pathol.

